# Devenir néonatal immédiat de la grande et l'extrême prématurité: données rétrospectives d'une unité de néonatalogie à Yaoundé, Cameroun de 2009 à 2013

**DOI:** 10.11604/pamj.2015.20.321.5289

**Published:** 2015-04-06

**Authors:** Anne Esther Njom Nlend, Cécile Zeudja, Annie Nga Motaze, Moyo Suzie, Nsoa Lydie

**Affiliations:** 1 Service de Pédiatrie et Prévention Infantile, Centre Hospitalier d'Essos, Caisse Nationale de Prévoyance Sociale, Yaoundé, Cameroun; 2 Association Camerounaise d'Aide aux Personnes et Familles Affectées par le VIH/SIDA, Cameroun

**Keywords:** Extrême prématurité, devenir néonatal, données rétrospectives, Extreme prematurity, neonatal outcome, retrospective data

## Abstract

L'objectif est de notre étude de décrire la typologie de la prématurité et mesurer la survie hospitalière à court terme des grands et extrêmes prématurés dans un pays à ressources limitées (PRL). C'est une étude descriptive rétrospective. Données extraites du registre des admissions du service. Inclusions de tous les nouveau-nés admis dans le service durant la période, ayant un âge gestationnel annoncé ≤ 36 semaines et 6 jours et plus de 26SA, avec au moins deux paramètres présents: âge gestationnel et poids de naissance. Principaux paramètres mesurés: pourcentage de nouveau-nés sortants vivants selon le type de prématurité: tardive, grande ou extrême. Nous avons recensé 1015 prématurés dont 314 grands prématurés (GP) et 61 extrêmes prématurés (EP). Le taux de nouveau-nés sortant vivants était de 95% chez les prématurés tardifs, de 71% chez les grands prématurés et de moins de 23% chez les extrêmes prématurés. Avant 28 semaines, le taux de mortalité était de prés de 100% chez les grands ou extrêmes prématurés de moins de 1000g contre 64% chez les plus de 1000g. Chez les GP le taux de décès était de 13% chez les nés par césarienne vs 21% chez ceux nés par voie basse (p ≤ 0,01). Le taux de prématurité médicalement induite était faible dans l'ensemble et de 3% chez les prématurés extrêmes. En conclusion le taux de mortalité hospitalière des EP est préoccupant, le faible taux de prématurité médicalement induite urge au renforcement de la prévention et à la mise en place de collaboration obstétrico-pédiatrique.

## Introduction

Parmi les 132 millions de naissances annuelles au monde, environ 4 millions de nouveau-nés succomberont durant les 28 premiers de vie [1]. Cette mortalité néonatale qui survient principalement dans les pays à ressources limitées (PRL) est imputable à trois causes majeures que sont la prématurité, l'infection et l'asphyxie néonatale. Si la létalité induite par la prématurité demeure importante dans les PRL, des progrès significatifs ont été faits pour la réduire dans les pays avancés, tant la grande prématurité que l'extrême prématurité [2–4]. Avec un taux de mortalité néonatale de 36 p1000 [5], le Cameroun fait partie des pays à fort taux de mortalité néonatale au monde. Quoique certaines équipes locales se soient penchées sur les causes des décès néonataux [6], la survie du grand prématuré dans notre contexte est mal connue. Aussi, dans le cadre de la mise en oeuvre d'un projet qui à améliorer la survie du nouveau-né dans la ville de Yaoundé autour du centre Hospitalier d'ESSOS agissant comme pôle de référence, il nous a paru nécessaire de mesurer la survie des grands prématurés admis dans notre service sur une période consécutive d'environ 5 ans.

## Méthodes

**Type d’étude:** il s'agit d'une étude descriptive rétrospective.

**Site de l’étude:** le site de l’étude est l'unité de néonatalogie du Centre Hospitalier d'ESSOS à Yaoundé. Le plateau technique à savoir le pole d'accueil, l'organisation des soins, le personnel fixe et la présence de pédiatres temps plein de jour assurant des astreintes nocturnes classe cette structure en unité de néonatalogie de niveau 2.

**Population d’étude:** la population d’étude a concerné les nouveau-nés admis dans l'unité entre Janvier 2009 et Décembre 2013 et répondant aux critères suivants: âge gestationnel annoncé inférieur ou égal à 32 semaines quelque soit le poids néonatal.

**Protocole de prise en charge:** le protocole de prise en charge standard à l'admission se décline comme suit: réchauffement en incubateur fermé ou table chauffante, mise à jeun durant les 24 premières de vie et perfusion à base de sérum glucosé à 10% et de gluconate de Calcium, oxygénothérapie par masque nasal en cas d'indice Silvermann supérieur à 3, antibiothérapie quasi systématique à base de céphalosporines de troisième génération et d'aminosides, traitement des apnées par aminophylline injectable, nutrition entérale par gavage discontinu à la tulipe dès le sevrage de l'oxygénothérapie. Chez les mères allaitantes une proposition de soins Kangourou est faite des que le nouveau-né est stable. Les autres prescriptions spécifiques sont faites sur appel de symptômes.

**Collecte des données:** les données présentées dans le cadre de ce travail ont été extraites du registre des admissions du service pour la période concernée et transférées dans un fichier Excel. Nous avons procédé à une analyse des principaux indicateurs suivants: taux de décès/survie brut pour les grands prématurés et les extrêmes prématurés. Ces indicateurs ont été exprimés en% avec leur intervalle de confiance (IC) à 95%. Les valeurs quantitatives médianes ont été exprimées avec leur rang interquartile. Une analyse comparative des décès utilisant le logiciel R version 3.0, avec le poids de naissance pris comme variable explicative à été faite. Etait considérée comme significative toute différence ayant une p-value ≤0,05.

**Considérations administratives et éthiques:** ce travail a reçu l'approbation du comité institutionnel d’éthique du Centre Hospitalier d'ESSOS ainsi que l'autorisation administrative de l'hôpital.

**Définition des termes:** grande prématurité: naissance comprise entre 28 et < 32 semaines d'aménorrhée; extrême prématurité: naissance avant 28 semaines d'aménorrhée; la survie à court terme était définie par le fait que le nouveau-né sorte vivant de notre unité quelque soit l’âge post menstruel ou post natal.

## Résultats

**Population d’étude:** durant cette période, 1015 prématurés ont été suivis dans le service parmi lesquels 318 étaient annoncés d’âge gestationnel ≤ 32 semaines dont 61 prématurés extrêmes. Le poids médian des extrêmes prématurés était respectivement de 1100g, (rang interquartile 900-1280); avec un terme médian de 26 semaines (rang interquartile 26-27); le poids des grands prématurés étaient de 1400g et le terme médian de 29 semaines.

**Figure 1:**
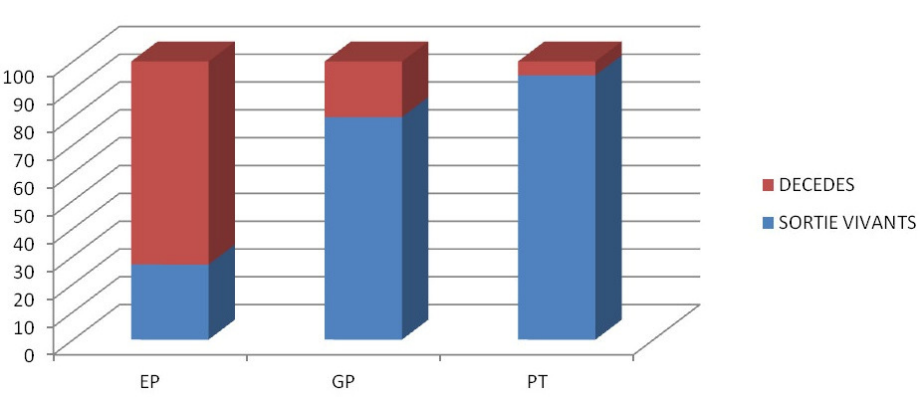
Pourcentage des prématurés sortis vivants dans l'unité de néonatologie du centre hospitalier d'Essos/CNPS entre 2009 et 2013 EP: extreme prématués; GP: grands prématurés; PT: premature Tardifs

**Survie globale à court terme selon le type de prématurité:** 176 nouveau-nés vont décédés donnant une mortalité hospitalière brute de 17,3% (IC 95%: 15,1-19,7). Le taux de prématurés tardifs sortant vivant était de 95%, il s'abaissait à 71% pour les grands prématurés et chutait à 23% chez les extrêmes prématurés p ≤ 0.001 (Figure 1).

**Figure 2:**
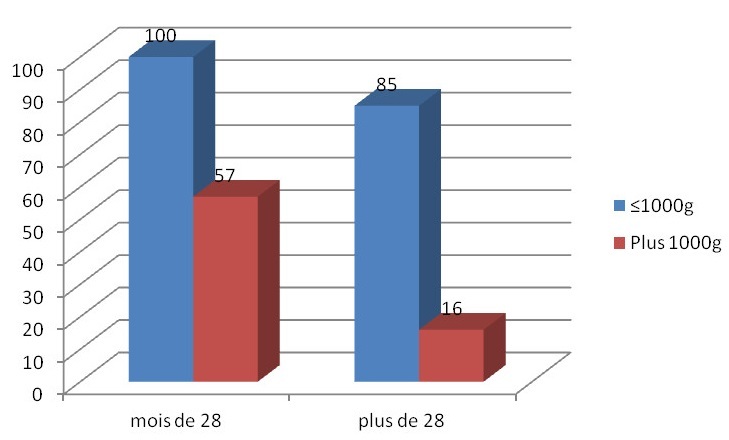
Pourcentage de grands et d'extrême prématurés décédés selon le poids de naissance au seuil de 1000g dans l'unité de néonatologie du centre hospitalier d’ Essos, Yaoundé

**Survie des grands et extrêmes prématurés selon le poids de naissance supérieur ou inférieur à 1000g:** selon les données de la Figure 2, un poids de naissance inférieur à 1000 g était associé à un plus grand risque de décès chez les extrêmes et grands prématurés respectivement 100% vs 57% avant 28 semaines et 57% vs 16^..^% après 28 semaines (p ≤ 0,001).

**Figure 3:**
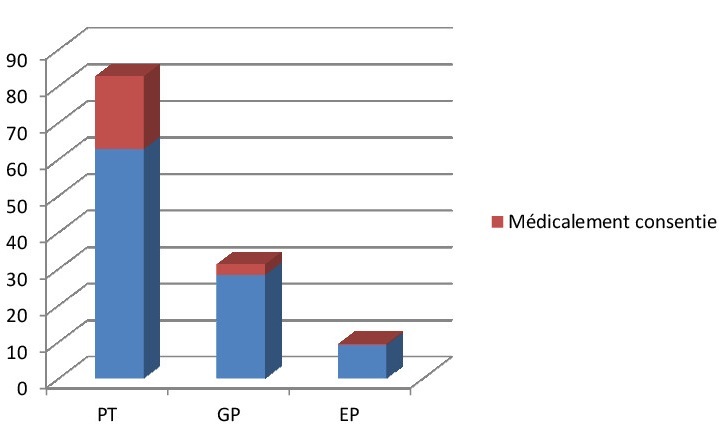
Taux d'extraction provoqué selon la catégorie de la prématurité au centre hospitalier d'Essos entre 2009 et 2013 (EP: extreme prématués; GP: grands prématurés; PT: premature Tardifs)

**Survie des extrêmes prématurés selon le mode d'accouchement:** le taux de mortalité intra-hospitalière était significativement bas en cas de césarienne (13%) vs 23% en cas d'accouchement par voie basse (p≤.0,01) et surtout le taux de prématurité induite par césarienne était très faible, voir Figure 3.

## Discussion

Ce travail préliminaire est intéressant, car peu de données sont disponibles sur la survie des grands prématurés dans les PRL d'Afrique subsaharienne. Les principaux résultats suggèrent que le taux de mortalité hospitalière des grands prématurés est encore significativement élevé à Yaoundé et ce, de manière outrageuse chez les extrêmes prématurés. Les chiffres sont à peine surprenant et appréciables en comparaison à des résultats observés dans des environnements similaires [7] Les données suggèrent de reconsidérer le seuil de viabilité historique de 28 semaines y compris dans les PRL, seuil rabaissé par l'OMS à 22 semaines et plus de 500g et à 25-26 semaines dans beaucoup de pays du Nord, après définition d'une zone dite grise [8, 9]. D'où la nécessité de définir des lignes directrices précises dans les PRL pour les moins de 28 semaines avec des protocoles extrêmement simplifiés [10] excluant toute forme d'acharnement sachant que, dans cette population sans réanimation avancée plus de 20% de nouveau-nés qui sortent vivant, cas de notre site. L'autre point source d'intérêt, qui devrait sans doute améliorer les chiffres de survie est la quasi absence de prématurité médicalement induite avant 28 semaines, suggérant comme cause majeure des grossesses non suivies. Ceci est d'autant plus préoccupant que dans cette population, les standards recommandent l'induction chez le prématuré de moins de 33 semaines. Ce faible taux de prématurité médicalement induite est également le résultat probable d'une faible collaboration obstétrico-pédiatrique et rend urgent la mise en place de réseau périnatal mieux organiser le transfert in utero et la gestion de ces nouveau-nés à haut risque.

## Conclusion

Nos travaux confirment la mortalité encore très importante de la grande prématurité et l'extrême prématurité dans les PRL ainsi que le faible taux de prématurité médicalement induite et suggèrent l'urgence à renforcer le suivi prénatal des grossesses et de mettre en place un réseau périnatal.
